# Behaviorally-determined sleep phenotypes are robustly associated with adaptive functioning in individuals with low functioning autism

**DOI:** 10.1038/s41598-017-14611-6

**Published:** 2017-10-27

**Authors:** Simonne Cohen, Ben D. Fulcher, Shantha M. W. Rajaratnam, Russell Conduit, Jason P. Sullivan, Melissa A. St Hilaire, Andrew J. Phillips, Tobias Loddenkemper, Sanjeev V. Kothare, Kelly McConnell, William Ahearn, Paula Braga-Kenyon, Andrew Shlesinger, Jacqueline Potter, Frank Bird, Kim M. Cornish, Steven W. Lockley

**Affiliations:** 1 Monash Institute of Cognitive and Clinical Neurosciences, School of Psychological Sciences, Monash University, Melbourne, Australia; 2 Division of Sleep and Circadian Disorders, Brigham and Women’s Hospital, Boston, USA; 3 Division of Sleep Medicine, Harvard Medical School, Boston, USA; 4 School of Health Sciences, Royal Melbourne Institute of Technology, Melbourne, Australia; 5 Boston Children’s Hospital, Boston, USA; 6 New York University Langone Medical School, New York, USA; 7 New England Center for Children, Southborough, USA; 8 Melmark New England, Andover, USA

## Abstract

Despite sleep disturbance being a common complaint in individuals with autism, specific sleep phenotypes and their relationship to adaptive functioning have yet to be identified. This study used cluster analysis to find distinct sleep patterns and relate them to independent measures of adaptive functioning in individuals with autism. Approximately 50,000 nights of care-giver sleep/wake logs were collected on school-days for 106 individuals with low functioning autism (87 boys, 14.77 ± 3.11 years) for 0.5–6 years (2.2 ± 1.5 years) from two residential schools. Using hierarchical cluster analysis, performed on summary statistics of each individual across their recording duration, two clusters of individuals with clearly distinguishable sleep phenotypes were found. The groups were summarized as ‘unstable’ sleepers (cluster 1, *n* = 41) and ‘stable’ sleepers (cluster 2, *n* = 65), with the former exhibiting reduced sleep duration, earlier sleep offset, and less stability in sleep timing. The sleep clusters displayed significant differences in properties that were not used for clustering, such as intellectual functioning, communication, and socialization, demonstrating that sleep phenotypes are associated with symptom severity in individuals with autism. This study provides foundational evidence for profiling and targeting sleep as a standard part of therapeutic intervention in individuals with autism.

## Introduction

Autism Spectrum Disorder (or autism) is a complex developmental disorder characterized by deficits in social-communication and repetitive and stereotyped interests and behaviors^1^. Sleep of children with autism is commonly disrupted, with 40–80% of children experiencing sleep problems compared with 25–40% in typically developing children^2,3^. Previous research has demonstrated a range of sleep difficulties among children with autism, including reduced total sleep time, delayed sleep onset, early sleep offset, and increased night awakenings^4,5^. Currently, there is a lack of understanding about the relationship between sleep difficulties and clinical symptoms in individuals with low functioning autism, defined as individuals with a severe intellectual, communication, and socialization impairment.

It is likely that the development and persistence of sleep difficulties in children with autism is multi-factorial. Pre-disposing factors – including abnormalities in comorbid medical^6^ and psychiatric^7,8^ conditions, and an imbalance in melatonin levels^9^– have been suggested as causes of sleep disturbances in children with autism. Precipitating events, such as family sleeping practices^10^ and emotional and behavioral difficulties^11^, are also known to disrupt the sleep-wake cycle in autism. Further, medications administered to decrease challenging behaviors in children with autism have been shown to affect sleep, increase existing sleep problems, and induce fatigue, insomnia and/or sedation^12^. Further exploration of factors that play a role in the etiology of sleep problems in autism may be beneficial for the development of treatment strategies to remediate sleep disturbance in individuals with autism.

Prior research has shown consistently that sleep problems are associated with core autism symptoms^13,14^, including social deficits, communication impairments, and repetitive and stereotyped behaviors^15,16^. Studies in the field to date, however, are primarily cross sectional and have focused on individuals with high functioning autism, who have the ability to communicate vocally and can tolerate actigraphic and polysomnographic (PSG) measurements of sleep^17–19^. While other studies have focused on the entire range of autism severity using subjective parent report measures^20,21^, the nature and prevalence of sleep difficulties specifically in individuals with low functioning autism remains understudied. Studies have suggested that the severity of sleep disruption increases with the severity of autism symptoms, but the extent of the sleep disruption remains unexplored. A cross-sectional study by Tudor *et al*.^22^ found that the severity of sleep problems (such as sleep onset delay and sleep duration) as defined by the Children’s Sleep Habits Questionnaire increased with the severity of autism symptoms (such as communication deficits) as defined by the Gilliam Autism Rating Scale^22^. Moreover, a cross-sectional study by Adams *et al*.^23^ using autism and sleep assessment batteries similarly found that autism severity was associated with an increased likelihood of sleep problems^23^. Previous studies, however, have primarily focused on disruptive sleep phenotypes in autism such as sleep duration and night awakenings in autism^24^ but have not investigated more nuanced properties like sleep regularity (i.e., sleep continuity and night-to-night variability). It remains unknown, therefore, what types of sleep problems predict what types of impairments in individuals with low functioning autism.

Cluster analysis is a method for identifying homogeneous subgroups (or clusters) within a dataset. In the autism literature, previous studies have used cluster analysis to clarify diagnostic heterogeneity in the disorder, but have been limited to identifying subgroups of autism on the basis of core symptoms rather than sleep^25–27^. Therefore it remains unknown whether individuals with autism can be reduced to subgroups based on their sleep properties. Addressing this shortcoming, we used cluster analysis to identify clinically meaningful subgroups of individuals with low functioning autism that are related to distinct patterns of sleep difficulties. We then examined whether differences in sleep phenotypes were associated with differences in clinical symptoms (e.g., intellectual functioning and adaptive functioning). We tested the hypothesis that differences in sleep characteristics can predict differences in the severity of adaptive functioning and core clinical symptoms in low functioning autism.

## Methods

### Data

This study was approved by the Partners Healthcare Institution Review Board (USA), Children’s Hospital Boston Committee (USA), and the Monash University Human Research Ethics Committee (Australia). A waiver of consent was obtained to access de-identified clinical data from both residential facilities.

### Participants

The data were collected from Melmark New England (MNE) and the New England Center for Children (NECC), two residential schools in Massachusetts, USA that offer intense applied behavior analysis therapy for individuals with autism and other developmental disabilities. All participants with a diagnosis of autism were assessed according to the Diagnostic and Statistical Manual of Mental Disorders (DSM-IV)^28^ by a Pediatrician or Psychiatrist. The study inclusion criteria were residential pupils under the age of 18 years with low functioning autism, confirmed by having an Intellectual Quotient (IQ) ≤ 70, as measured by either the Leiter-R International Performance Scale-Third Edition^29^ (*n* = 18) or the Stanford-Binet Intelligence Scale-Fifth edition^30^ (*n* = 40); or impairments in communication, socialization, and adaptive functioning, as measured by the Vineland Adaptive Behavior Scales (VABS^31^ (*n* = 60). Missing IQ and VABS scores were due to individuals being unable to participate in standardized testing due to the extent of their impairments (i.e., severe deficits that could not be quantified by formal assessments), therefore these individuals were still included in the analysis.

Data were collected on 41 children (35 boys, 6 girls) with a mean age (±SD) of 14.56 ± 3.63 years from a total of 47 children who were resident at MNE from 2008 to 2013. Data were also collected on 65 children (52 boys, 13 girls, M = 14.91 ± 2.74 years) from a total of 132 children who were resident at NECC from 2010–2013. The 6 MNE residents and 67 NECC residents who were excluded from the study cohort were either considered to have an alternative diagnosis or were above 18 years of age. Eighteen years of age was considered a reasonable inclusion criterion for participants to be labeled as adolescents. The total sample consisted of 106 individuals with clinically diagnosed low functioning autism who were between 5 and 18 years old (87 boys, 14.77 ± 3.11 years). The data collection duration for each participant ranged from 0.5 to 5.9 years (2.2 ± 1.5 years, between February 2008 and November 2013) due to the variable admission dates into the facilities and each individual contributed between 42 to 1858 nights of data (734 ± 424 nights). Eighty percent of the participants were White (*n* = 94), and the remaining demographic sample included African American (*n* = 4), Hispanic (*n* = 2), Asian (*n* = 2) and individuals of unknown ethnicity (*n* = 4).

### Sleep-wake recordings

As a part of routine clinical care, trained care-givers on duty at both residential facilities were responsible for recording sleep-wake state from 19:00 to 7:00 h during frequent bed checks and making a judgment as to whether the participant was awake (defined as eyes open with bedroom activity) or asleep (defined as eyes closed with no bedroom activity). Sleep-wake behavior was measured every 30 minutes at MNE and every 15 minutes at NECC, and recordings were taken every night from initial entry into the residential facility. Participants residing at MNE had variable ‘lights-off’ times, which could change throughout their stay in the residential facility. Seven participants residing at MNE had prompted or unprompted toilet awakenings throughout the night due to incontinence, which was considered as time awake in this analysis. Participants at NECC had a standard fixed lights-off time of 21:00 h with no prompted night awakenings. Both schools had fixed lights on/wake times at 7:00 h. Bed-time routines were maintained on weekends at both residential facilities. A total of 77,838 nights of sleep-wake recordings were available from this dataset.

### Clinical information

Clinical information was obtained from the school databases, which included demographic information (such as age, gender), and clinical information as screened by a Psychiatrist of Pediatrician. The clinical information available from this dataset included, medical information (such as seizures, gastrointestinal disorders), psychological information (such as anxiety, attention deficit hyperactivity disorder), and medication information (including nights with and without medications). These variables (with the exception of age) were transformed into binary variables and were coded as a 0 (absence of characteristic) or 1 (presence of characteristic). Medications were grouped into drug classes that affect sleep (including SSRI,s, anti-histamines, anti-psychotics, melatonin, beta-blockers and benzodiazepines) and the proportion of nights on each medication class was calculated for each individual. Adaptive functioning scores were also available using VABS, which measures five domains: communication, daily living, socialization, motor skills, and maladaptive behavior. These domains make up an overall composite score of adaptive behavior^31^. The VABS has demonstrated adequate internal consistency reliability and good test-retest reliability with coefficients ranging between 0.80 and 0.90^31^. Both IQ and adaptive functioning were only assessed upon entry into the residential facility, but are considered to be a stable measure of developmental functioning across an individual’s lifespan^32^.

### Calculating sleep statistics and sleep features

Sleep parameters were computed from nightly observations of sleep and wake including: i) total sleep time, ii) sleep onset, iii) sleep offset, iv) sleep efficiency, and v) number of night awakenings (see Table 1 below for definition of parameters). These five sleep parameters were chosen, as they were the most accurate sleep measurements that could be calculated from this coarse temporal dataset. It is important to note that although the measurement of sleep in our sample may be less accurate than lab-based, controlled studies (such as sleep-lab or actigraphy studies), our dataset, which is unique in comprising an unprecedented volume of real world data, facilitates an ecologically valid analysis of sleep in this population. To ensure good data quality, nights containing any missing data points (missing 15–30 minute recordings, for example), or nights with no data (due to weekends or holiday breaks, for example) were excluded from our analysis. In total, 26% of nights (*n* = 20,484) were excluded due to missing data prior to performing sleep calculations. The remaining dataset consisted of 29,756 nights of data from individuals at MNE and 27,598 nights of data from individuals at NECC, yielding a total dataset containing 57,354 nights of sleep-wake data. These data included all the sleep-wake recordings for every individual across their available recording period. We also examined a restricted range of data across a 1-year recording period, however, to assess the stability of the cluster analysis results across time.

**Table 1:** Five sleep statistics used to summarize each individual night of sleep-wake data.

Sleep statistic	Definition
Total sleep time (h)	Total time spent asleep.
Sleep onset time (hh:mm)	Clock time at the start of the first episode of sleep.
Sleep offset time (hh:mm)	Clock time at the end of the last episode of sleep.
Sleep efficiency (%)	Total sleep time divided by the sleep interval (sleep offset − sleep onset).
Night awakenings (#)	Total number of awakenings recorded following the initiation of the sleep period. Each awakening had to be followed by an episode of sleep.

**Figure 1:**
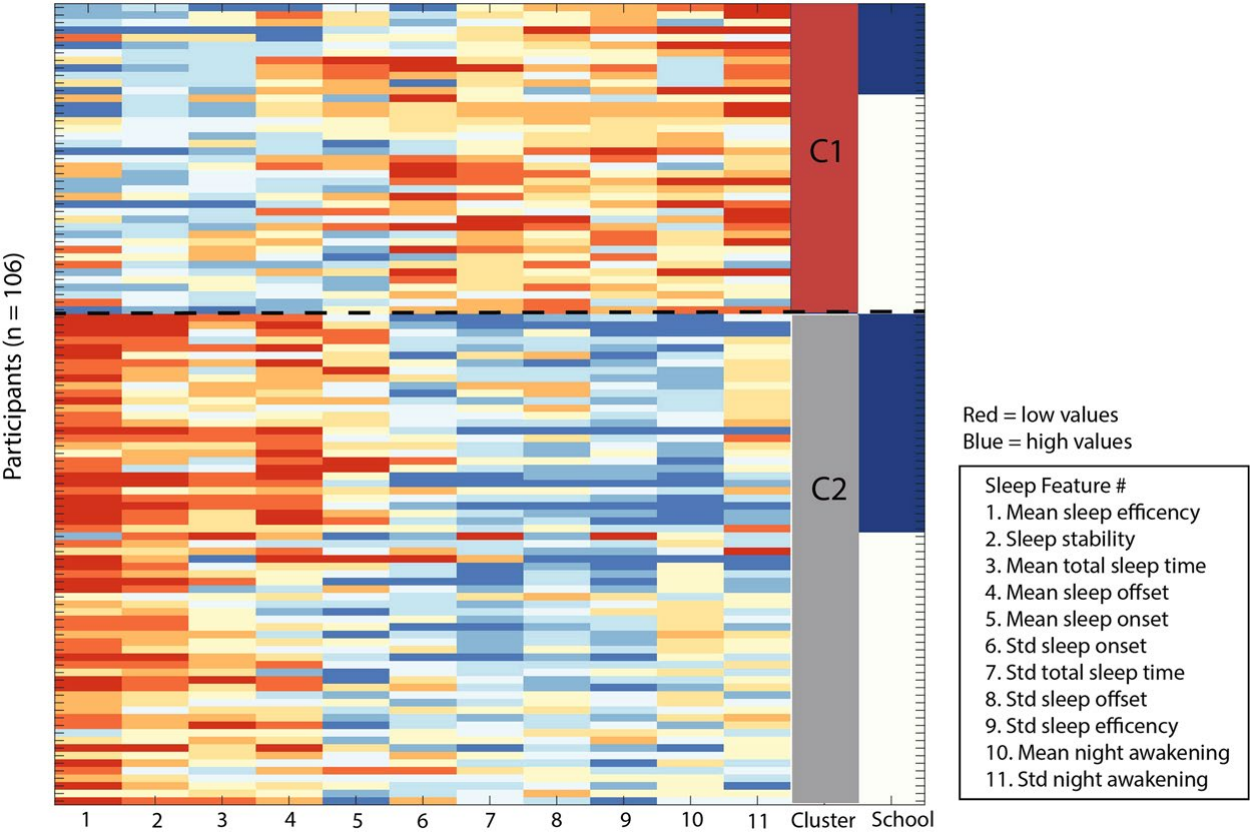
A visual representation of the sleep profiles for each individual across the 11 sleep features, including the cluster solutions. Elements in the sleep features matrix were normalized and visualized using color from blue (high values) to red (low values). Participants (rows) and sleep features (columns) were compared using squared Euclidean distances and were then reordered using Wards linkage clustering to place similar sleep features and similar participants close to one another. The results produced two distinct clusters; C1 (stable sleepers, *n* = 41) and C2 (unstable sleepers, *n* = 65), which is represented by the far right bar. The school number is indicated in the right bar with the white representing MNE and blue representing NECC. The sleep features are labeled 1–11 on the horizontal axis. Std = standard deviation.

To convert the above features of individual nights of sleep to summary statistics for individuals across their entire sleep period, we calculated the mean and standard deviation of each of the five sleep parameters, resulting in 10 sleep features (shown in the key for Fig. 1). In addition to these 10 sleep features, a measure of sleep regularity was included: the Sleep Regularity Index (SRI)^33^. This feature is based on the probability of an individual being in the same state (asleep or awake) at any two points 24 hours apart. Mathematically, the SRI was computed as $\frac{100}{T}{\sum }_{t}{S}_{t}\,\times \,{S}_{t+24}$, where *S* is a sleep indicator (1 for wake, −1 for sleep) and the sum over time *t* (hours) was taken over all *T* sleep observations with a matching observation 24 h later. *T* reflected the number of sleep observations across an individual’s whole recording period which varied between participants in each residential facility. The SRI takes a value between −100 and 100, where 100 indicates a perfectly regular nightly pattern of sleep and wake, that repeats exactly every night, 0 reflects an individual that wakes and sleeps at random each night, and −100 indicates a pattern that never matches with itself 24 hours later. This measure also accounts for variable bedtimes, as it was calculated from the start of the sleep episode. Thus a total of 11 sleep features were used to summarize the sleep of each participant across their full recording period (and across a one-year recording period to test for robustness). No pairs of sleep variables showed a strong linear dependence; all pairwise Pearson correlation coefficients between sleep variables had *R* ^*2*^ < 0.30.

### Cluster analysis

Prior to performing a cluster analysis, an outlier-robust sigmoidal normalizing transformation was applied to each sleep feature (as this transformation normalizes scores according to the median and interquartile range, iqr). Mathematically, this was computed as $\tilde{x}=\,(x-{\rm{median}}(x))/(1.35{\rm{iqr}}(x))$. This calculation adjusted for outliers in the data and allowed sleep features with different ranges to be compared meaningfully^34^. Hierarchical clustering was performed using Ward’s method with a Euclidean distance metric. To evaluate the optimal number of clusters in our data, we used several evaluation methods, including the Gap Statistic^35^, Silhouette method^36^, and Calinski-Harabrasz criterion^37^. The number of clusters, *k*, was determined as the value of *k* that was the most consistent across these evaluation methods.

Differences in sleep features (including total sleep time and sleep onset), clinical characteristics (including IQ and adaptive functioning), drug classes that impact sleep, and medical and psychological comorbidities (including anxiety, attention deficit hyperactivity disorder, gastro-intestinal disorders and epilepsy) between clusters were quantified using a Welch’s t-test, correcting for multiple comparisons using the method of Bonferroni. Univariate linear regression was used to evaluate the correlation of each participant’s 11 sleep features to each of the clinical variables (e.g., IQ, communication scores, socialization scores, daily living scores) separately to investigate whether any single sleep feature predicted any single clinical variable. The correlations between sleep features and clinical variables were only performed using individuals in sleep clusters that had clinical data such as IQ scores (*n* = 58) and adaptive functioning scores available (*n* = 60).

## Results

### Sleep phenotypes in children and adolescents with low-functioning autism

**Figure 2:**
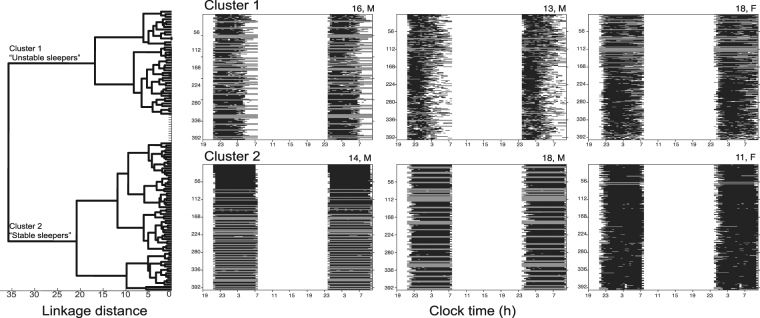
Hierarchical cluster analysis found two clusters of individuals within the sample: cluster 1 (*n* = 41) and cluster 2 (*n* = 65) as shown by the dendrogram (left), with participants in each cluster distinguished by their severity of sleep disruption as illustrated by a subset of sleep raster plots (right). A dendrogram (left), shows a visualization of the hierarchical cluster analysis and useful summary of the data. Each observation (or individual) is represented by a node placed on the vertical axis, and the horizontal axis (linkage distance) indicates the distance between observations in a cluster. A random selection of individual participant sleep raster within each sleep cluster is plotted to the right of the dendrogram, with their age and gender labeled above each raster. The raster plot shows a double plot of an individual’s sleep-wake behavior across their recording period, with black bars representing sleep and white representing wake behaviour across the recording period (~19:00–07:00 h). Missing data within the recording period are shown in grey. As seen, the unstable cluster had shorter and more variable total sleep time, more variability in sleep onset and sleep offset and greater night awakenings when compared to individuals in the stable sleep cluster.

Using a set of 11 sleep features, we performed hierarchical cluster analysis on all 106 individuals, as shown in Fig. 1. The results of the hierarchical cluster analysis show two robust clusters of individuals within the sample: cluster 1 (*n* = 41) and cluster 2 (*n* = 65). A two-cluster solution was consistent across a range of parameters controlling the hierarchical clustering, including distance metrics (i.e., Euclidean, Correlation, Spearman), linkage methods (i.e., Ward, Average, Weighted), and evaluation criterion (Gap statistic, Silhouette method and Calinski-Harabrasz criterion). Examples of individual raster plots for each cluster are shown in Fig. 2.

**Figure 3:**
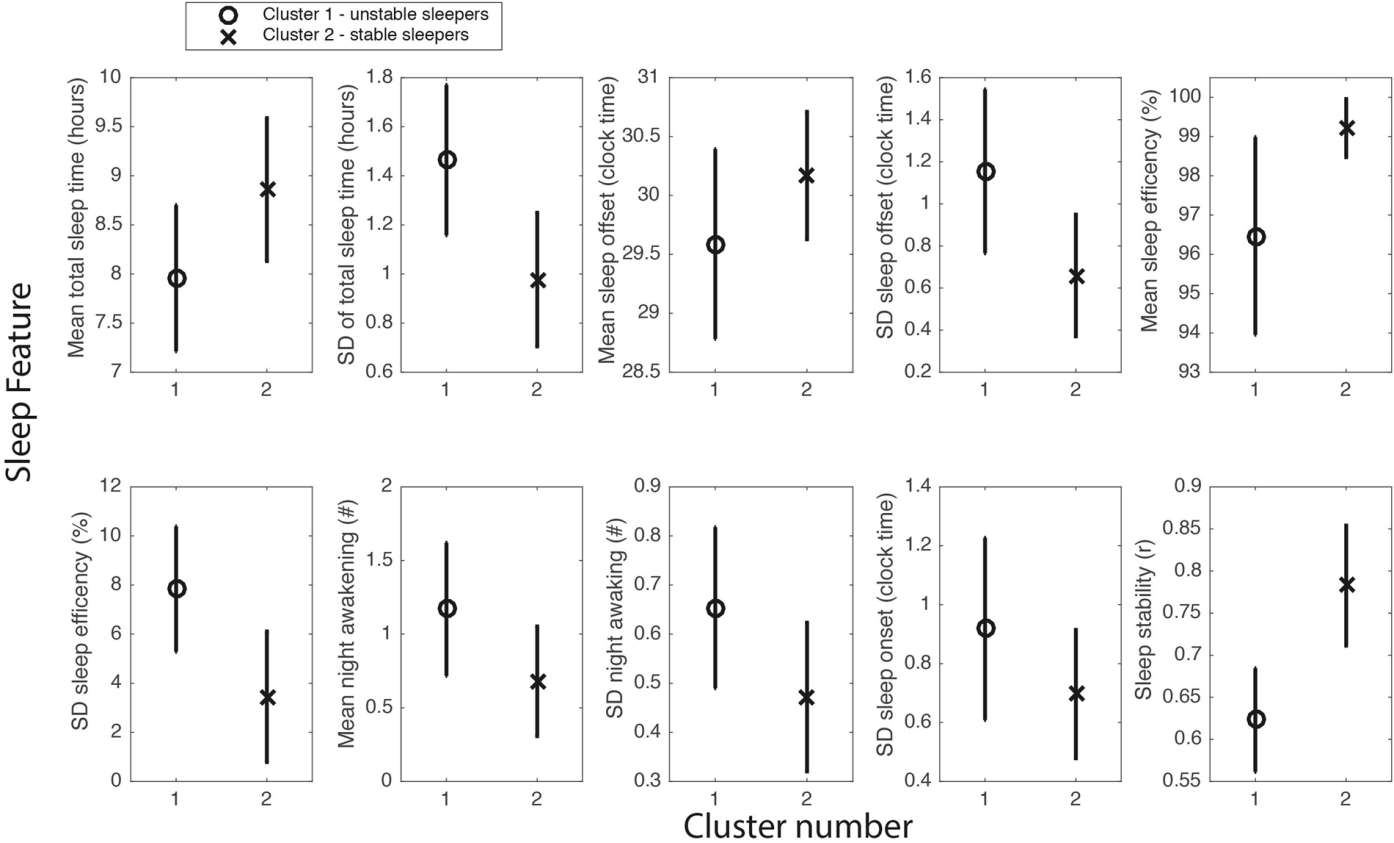
Sleep features that significantly distinguish the stable (*n* = 41) and unstable (*n* = 65) sleep clusters using Welsch’s t-tests. The means ± standard deviations are plotted for each cluster. *p < 0.05/11 (i.e., significance value/number of comparisons).

Welch’s t-tests found that cluster 1 and cluster 2 differed significantly in 10 of the 11 sleep features (*p* < 0.004) (see Fig. 3 for 10 of the 11 sleep features that were significant). Participants in cluster 2 have significantly longer and less variable total sleep time (TST), reduced variability in sleep onset, later and less variable sleep offset, increased sleep regularity, higher and less variable sleep efficiency, and fewer night awakenings when compared to participants in cluster 1. Cluster 1 is thus characterized by sleep features that indicate highly variable and unstable sleep and therefore this group was given the label ‘unstable’ sleepers relative to cluster 2, who were labeled ‘stable’ sleepers. See supplementary Fig. 1 and Fig. 2 for additional samples of participants sleep-wake raster plots within each sleep cluster.

To investigate the robustness of the clusters, we repeated the cluster analysis for participants in each school separately. Again, hierarchical clustering and evaluation procedures consistently indicated a two-cluster solution within each school. The clusters obtained in each school individually matched the same characteristics found in the full clustering as described above, allowing us again to use the ‘stable’ and ‘unstable’ labels to describe the two clusters. Eleven participants at MNE (26.3%) and 29 participants at NECC (44.6%) matched an ‘unstable’ sleep profile when compared to 30 participants at MNE (73.1%) and 36 participants at NECC (55.36%) who matched a ‘stable’ sleep profile. We also examined the reproducibility of cluster solutions by deriving a cluster solution from MNE and applying it to individuals at NECC (and vice versa). The predicted cluster accuracy for MNE (based on cluster solution from NECC) was 73%, and the predicted cluster accuracy from NECC (based on cluster solution from MNE) was 68%. Thus, in approximately 70% of cases, we can predict cluster assignments from one school and apply it another school, highlighting the stability of cluster solutions. Furthermore, similar results were obtained when examining an individual’s sleep-wake recording over a 1-year period suggesting stability of cluster membership for an individual across time.

### Characterizing and evaluating sleep phenotypes

**Figure 4:**
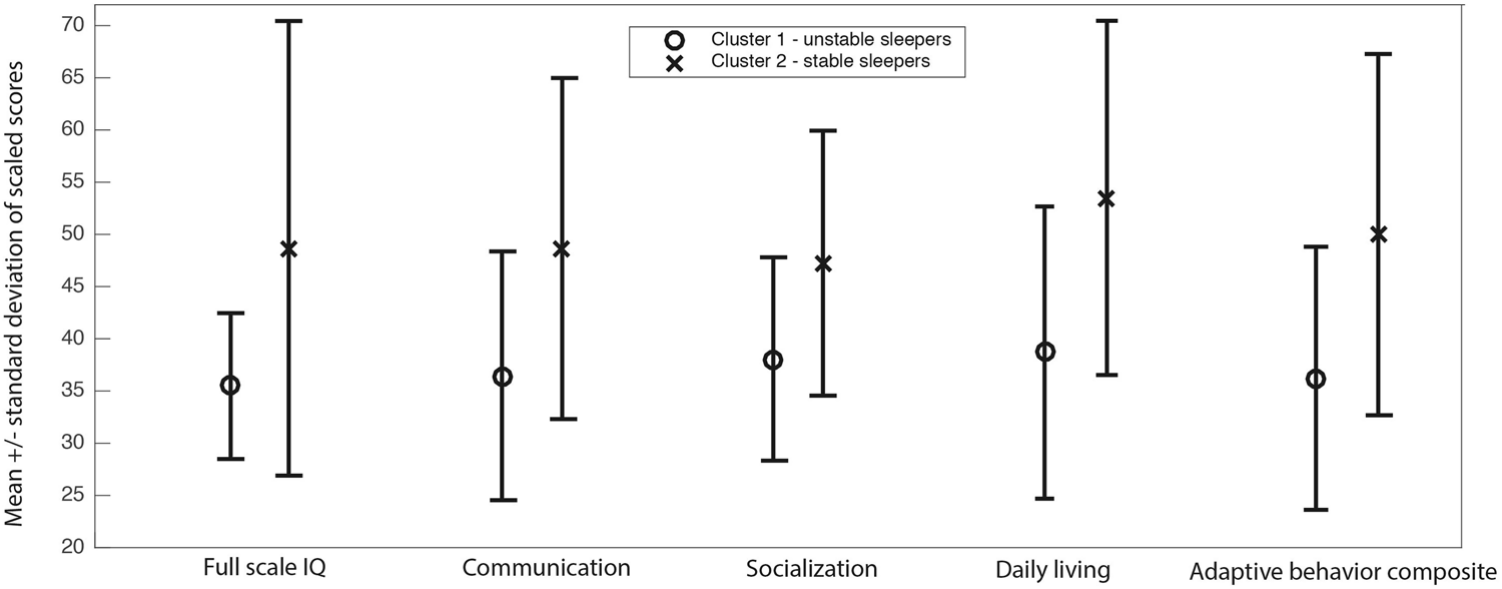
The two sleep groups were distinguished by scores that determine adaptive functioning in autism. Profile of scaled scores for intellectual quotient (IQ) and Vineland Adaptive Behavior Scales parent ratings of communication, socialization, daily living and adaptive behavior composite across the distinct two-sleep phenotypes *p < 0.05/5 (i.e., significance value/number of comparisons).

Next we investigated whether the individuals in the two sleep clusters were distinguished by their clinical, medical, and metadata scores (data that was *not* used to cluster individuals). Welch’s t-tests, adjusted for multiple comparisons using Bonferroni, were used to investigate differences in sleep clusters on all clinical information provided. The ‘unstable’ sleep group had significantly reduced full-scale IQ scores, communication scores, daily living and overall adaptive behavior composite scores when compared to participants in the ‘stable’ sleep group (see Fig. 4 where all results *p* < 0.01). No significant differences between clusters were found for age, gender, psychiatric comorbidities, and medical comorbidities. Welch’s t-tests corrected for multiple comparisons also found no significant differences in the portion of children from each cluster using drug classes (including SSRI’s, anti-convulsant, anti-psychotics) that are known to affect sleep (*p* > 0.06).

Finally, univariate linear regression was used to evaluate the correlation of each participant’s sleep features and their clinical features such as IQ and adaptive functioning scores. All individuals exhibited a small correlation (*R* ^2^ ≤ 0.04) between each of the 11 sleep features (when taken individually) and IQ, socialization, communication and daily living scores, suggesting that no individual sleep features can explain the differences uncovered by cluster analysis. This demonstrates the benefit of using a multivariate cluster analysis that simultaneously incorporates a combination of sleep features to determine clinically informative sleep phenotypes, rather than a single sleep feature (such as total sleep time).

## Discussion

This study is the first to report robust clusters of sleep behavior in children with low functioning autism using an unprecedented dataset of over 50,000 nights of sleep. We report two distinct sleep phenotypes, labeled ‘stable’ and ‘unstable’ sleepers each with characteristic differences in the severity of key sleep features. Notably, the ‘unstable’ sleepers displayed greater impairments in functioning, including lower IQ scores as well as adaptive functioning scores, when compared to the ‘stable’ sleep group. The same qualitative findings were obtained when considering individual schools separately, and these findings were independent of age, gender, and medication administration. The findings from this study coincide with previous research suggesting that sleep deficits increase with autism symptom severity (e.g., Tudor *et al*.^22^; Adams *et al*.^23^), and alongside recent work by Palmer *et al*.^25^ and Kitazoe *et al*.^27^ highlights the utility of cluster analysis in the clinical sub-typing of autism. The implications of this study are significant, as it identifies stable and unstable sleep profiles in autism, and links robust changes in sleep behavior to adaptive functioning in individuals with low functioning autism. Moreover, this study draws attention to sleep patterns over time, and demonstrates how sleep continuity (not just hours of sleep or night time awakenings) is associated with adaptive functioning in autism.

Clinical subtyping has been defined as one of the major short-term barriers in autism research, given the challenge of clinical heterogeneity in autism^38^. Previous studies have been unable to capture distinct sleep profiles in autism, as they have primarily used cross-sectional data from individuals with high functioning autism, and have limited their measurements to parent reports, or small PSG and actigraphy studies^39,40^. Current objective sleep measures pose methodological challenges, as individuals with autism often have difficulty tolerating these tools due to sensory sensitivities^41^. Moreover, a recent study has shown that infrequent random parental observations of sleep are correlated with actigraphy measured sleep duration^42^, which challenges the notion that sleep recordings need to be objectively measured in order to be validated. The nature of our dataset (longitudinal observations over a period of 5 years and the ability to extract a large number of sleep characteristics from each individual) allowed us to identify distinct patterns of sleep-wake behavior in individuals with low functioning autism with ecological validity. We also incorporated new measures of variability such as sleep regularity, which allowed us to measure subtle properties of sleep-wake behavior, including sleep timing. Further investigation using these representative features could provide new insights into the sleep profiles of individuals with sleep disorders, or high functioning autism. Although the present findings will require more complete assessments and independent replications, they suggest that cluster analysis may be able to partition individuals with low functioning autism into clinically meaningful subgroups on the basis of their sleep-wake behavior.

### Limitations and future directions

This study should be viewed as an initial step towards the identification of sleep phenotypes using cluster analysis in low functioning autism, and as such, our results need to be interpreted in the context of the data acquisition. Sleep-wake recordings were based on discrete 15–30 minute observations – an approach that differs from the continuous recordings obtained from actigraphy or PSG. Hence, the reliability and validity of the observations poses a limitation, in addition to the accuracy of sleep measurements, which are based on a coarse time-sampling approach. We acknowledge that although the quality of sleep measurement in our study is less accurate than traditional lab-based studies using objective measures such as actigraphy, this limitation was counterbalanced by the uniqueness of this dataset in that it comprises an unprecedented volume of real world data. This study focused on a relatively small and understudied population of individuals with low functioning autism, which may not be generalizable to outpatient autism samples, who are not living in residential care, nor children with high functioning autism. Further validation of independent cohorts, including a wider variety of demographics, greater sample sizes, outpatient samples, and levels of autism severity is needed to allow for generalizability of findings. While night awakenings from MNE were not taken into account in this analysis, the same consistent sleep clusters were evident in both schools, suggesting that sleep clusters were not systematically biased by artificial night awakenings. Furthermore, adaptive functioning test results (i.e., VABS scores) were only determined at one point in time when participants were initially enrolled at the residential facilities, when their autism symptoms were potentially most severe. While it is possible that later intervention may have moderated these scores, and therefore the association between sleep phenotypes and adaptive functioning, this is unlikely given the same sleep phenotypes were evident when performing cluster analysis of individual sleep recordings across a one-year recording period. Future studies examining sleep patterns could examine sleep profiles and adaptive behavior data at concurrent time points, in order to validate this finding and assess effects of any clinical interventions. Thus, the timing and type of clinical assessments used in this study present a potential limitation. Lastly, we acknowledge that the clusters identified using this method are based on a group effect, and while individual variability between participants exists, this method does not characterize individual sleep phenotypes. While this dataset contained a wide range of ages, demographic characteristics, medications, and recording periods, the robustness of our results validate our assumption that this large volume of detailed data is sufficient to overcome its limitations.

### Implications of findings

Overall, the findings of this study suggest a deeper relationship between sleep and autism than has previously been acknowledged. We used over 50,000 nights of data to uncover two distinct sleep phenotypes with associated differences in symptomatic profiles. The identification of distinctive sleep profiles in individuals with low functioning autism creates a foundation for the development of enhanced diagnostic algorithms and the potential to offer more personalized therapies in the future that will hopefully improve long-term outcomes for the nearly 1 in 68 individuals affected by this pervasive developmental disorder^43^.

## Electronic supplementary material

Supplementary data
