# Supplementary material for: Behaviorally-determined sleep phenotypes are robustly associated with adaptive functioning in individuals with low functioning autism
Source: Sci Rep. 2017 Oct 27;7:14228. doi: 10.1038/s41598-017-14611-6 (PMC5660229; doi:10.1038/s41598-017-14611-6)
Supplement: Supplementary file 1 — Supplementary data [file 41598_2017_14611_MOESM1_ESM.pdf]

Behaviorally-determined sleep phenotypes are robustly associated with adaptive functioning in individuals with low functioning autism

Simonne Cohen, PhD1, Ben D. Fulcher, PhD1, Shantha M. W. Rajaratnam, PhD1,2,3, Russell Conduit, PhD4, Jason P. Sullivan2, Melissa A. St Hilaire, PhD2,3, Andrew Phillips2,3, Tobias Loddenkemper, MD3,5, Sanjeev V. Kothare, MD3,5,6, Kelly McConnell, PhD7, William Ahearn, PhD7, Paula Braga-Kenyon, PhD7,8, Andrew Shlesinger8, Jacqueline Potter, PhD8, Frank Bird, M.Ed8, Kim M. Cornish, PhD1, *Steven W. Lockley, PhD1,2,3

1.Monash Institute of Cognitive and Clinical Neurosciences, School of Psychological Sciences, Monash University, Melbourne, Australia.
2.Division of Sleep and Circadian Disorders, Brigham and Women's Hospital, Boston, USA.
3.Division of Sleep Medicine, Harvard Medical School, Boston, USA.
4.School of Health Sciences, Royal Melbourne Institute of Technology, Melbourne, Australia.
5.Boston Children's Hospital, Boston, USA.
6.New York University Langone Medical School, New York, USA.
7.New England Center for Children, Southborough, USA.
8.Melmark New England, Andover, USA.

Address correspondence to: Steven W. Lockley, Ph.D., Division of Sleep and Circadian Disorders, Brigham and Women's Hospital, 221 Longwood Avenue, Boston, MA 02115, USA, [slockley@hms.harvard.edu]

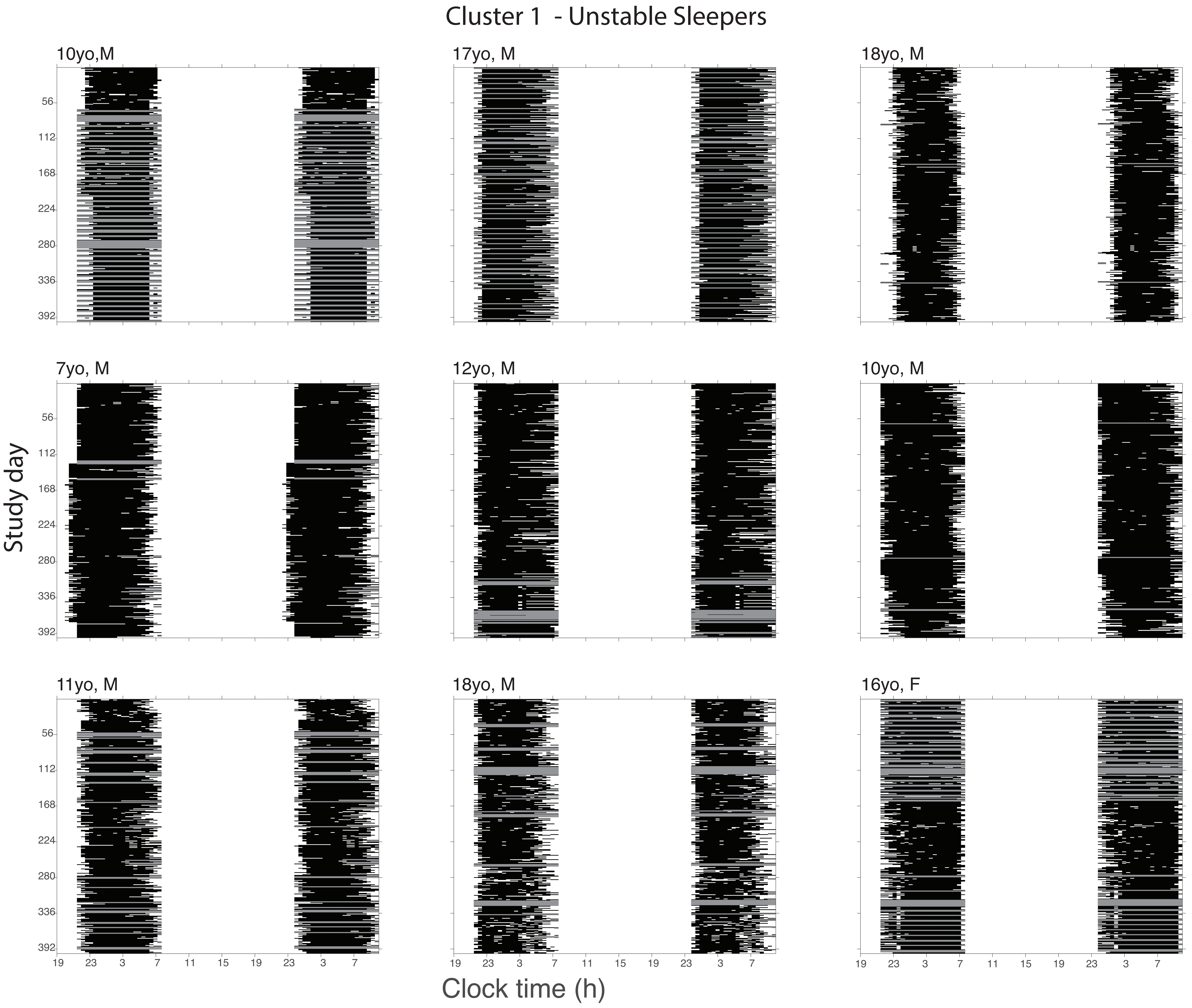

Figure 1: A random subtest of individuals within the unstable sleep cluster. A random selection (n = 9) of individual participant sleep rasters within the unstable sleep cluster is plotted, with their age and gender labeled above each raster. The raster plot shows a double plot of individuals' sleep-wake behavior across their recording period, with black bars representing sleep and white representing wake behavior across the recording period (~19:00-07:00h). Missing data within the recording period are shown in grey. As seen, the unstable sleep cluster had more variable sleep patterns with later sleep onset, more night awakenings, and poorer sleep stability.

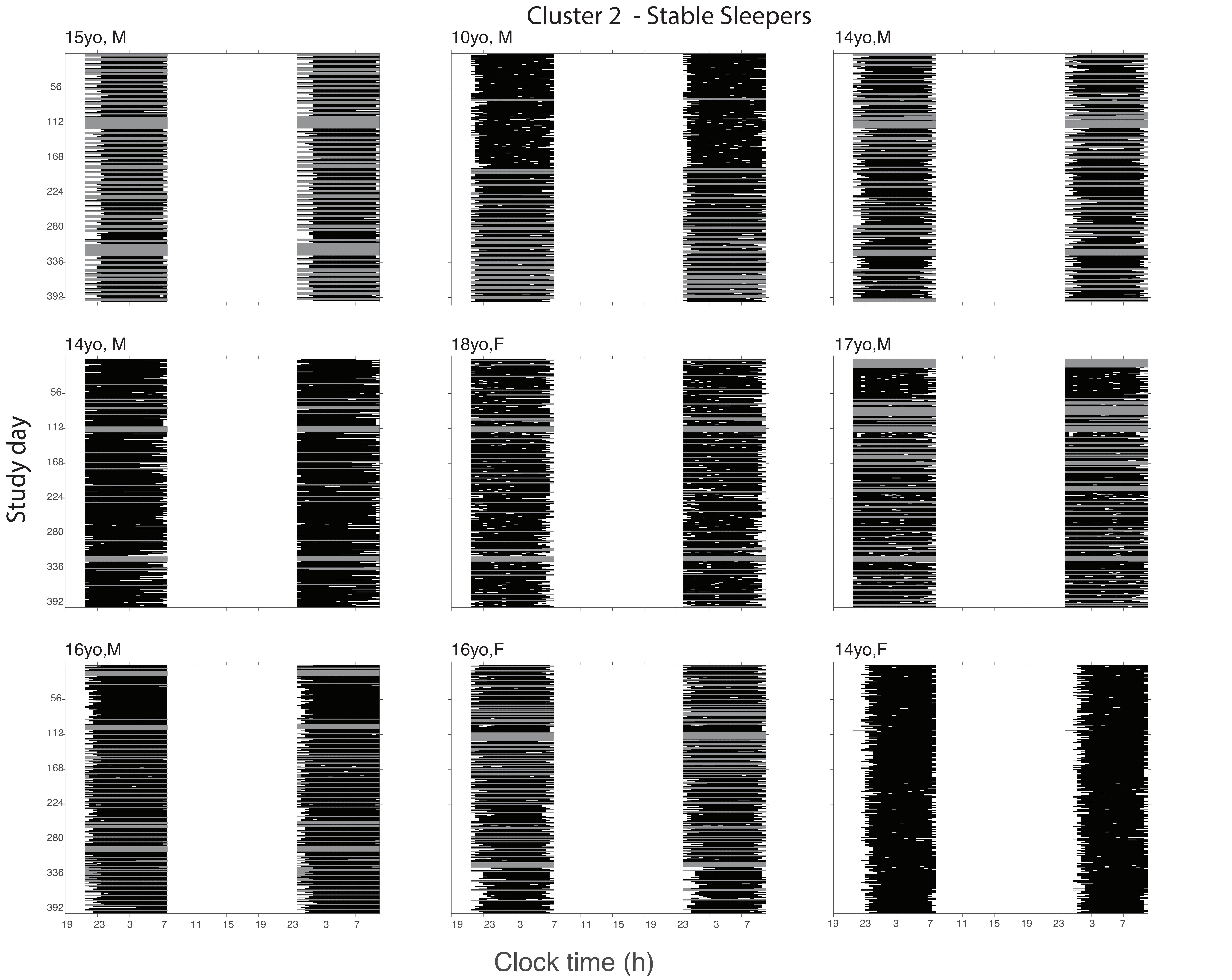

Figure 2: A random subtest of individuals within the stable sleep cluster. A random selection (n = 9) of individual participant sleep rasters within the stable sleep cluster is plotted, with their age and gender labeled above each raster. The raster plot shows a double plot of individuals' sleep-wake behavior across their recording period, with black bars representing sleep and white representing wake behavior across the recording period (~19:00-07:00h). Missing data within the recording period are shown in grey. As seen, the stable sleep cluster had more consistent sleep patterns with less night awakenings, and more stable sleep across time.
